# Supplementary material for: Ferroptosis Inhibitory Compounds from the Deep-Sea-Derived Fungus Penicillium sp. MCCC 3A00126
Source: Mar Drugs. 2023 Apr 10;21(4):234. doi: 10.3390/md21040234 (PMC10144380; doi:10.3390/md21040234)
Supplement: Supplementary file 1 [file marinedrugs-21-00234-s001.zip › marinedrugs-2113175-supplementary.pdf]

YJA-40 C 8mg H

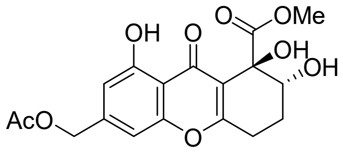

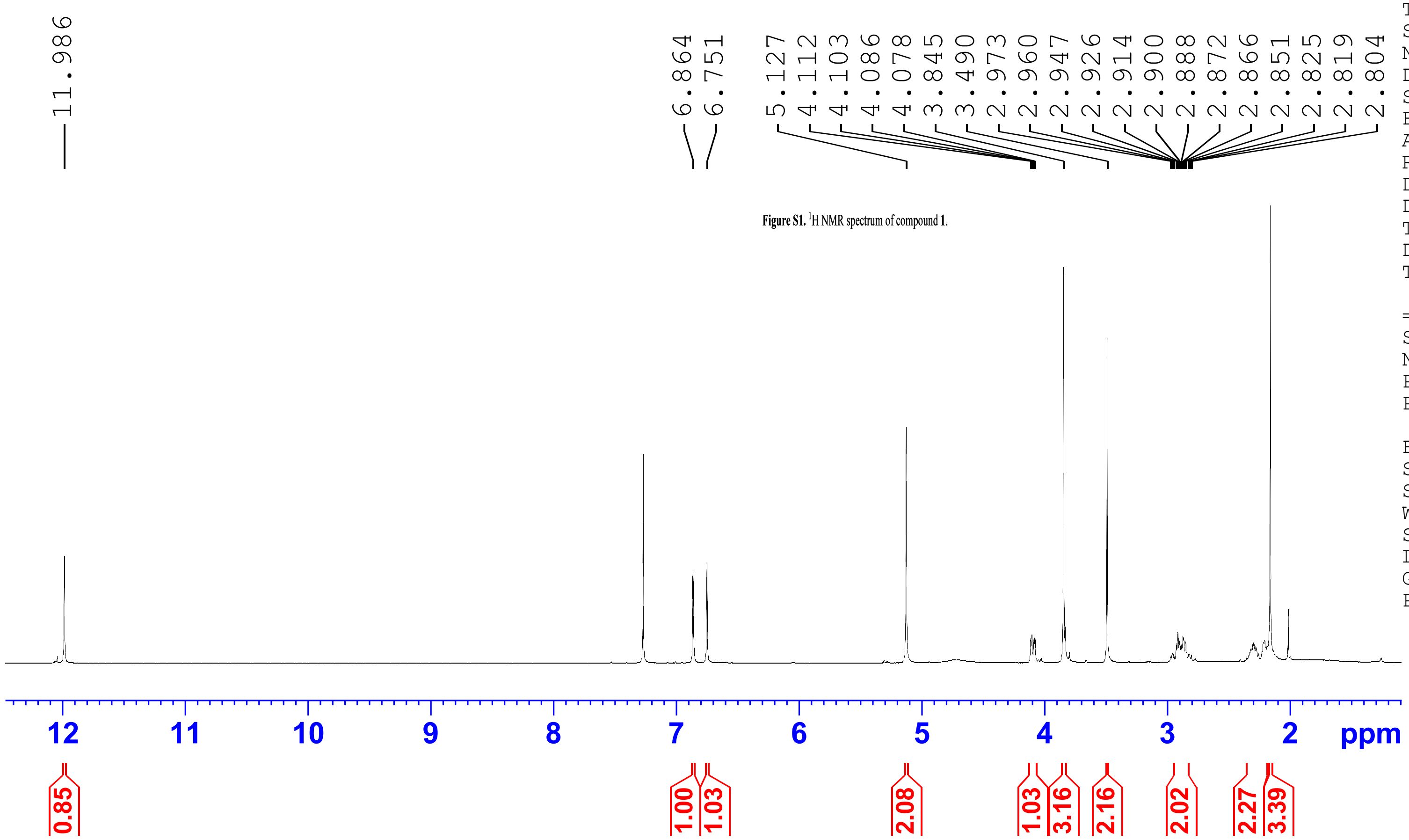

Figure S1. ^{1}H NMR spectrum of compound 1.

```
Current Data Parameters
NAME        YJA-40 C 8mg
EXPNO                  1
PROCNO                 1

F2 - Acquisition Parameters
Date_           20210912
Time               17.27
INSTRUM            spect
PROBHD   5 mm PABBO BB-
PULPROG             zg30
TD                 65536
SOLVENT            CDCl3
NS                    18
DS                     2
SWH             8012.820 Hz
FIDRES          0.122266 Hz
AQ             4.0894465 sec
RG                   203
DW                62.400 usec
DE                  6.50 usec
TE                 296.3 K
D1            1.00000000 sec
TD0                    1

======== CHANNEL f1 ========
SFO1      400.1324710 MHz
NUC1                   1H
P1                  13.90 usec
PLW1          12.14299965 W

F2 - Processing parameters
SI                 32768
SF         400.1300056 MHz
WDW                   EM
SSB                    0
LB                  0.30 Hz
GB                     0
PC                  1.00
```

Figure S1. ^{1}H NMR spectrum of compound **1**.

YJA-40 C 8mg C

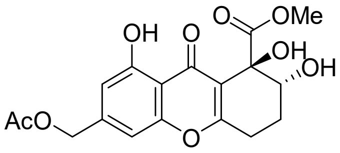

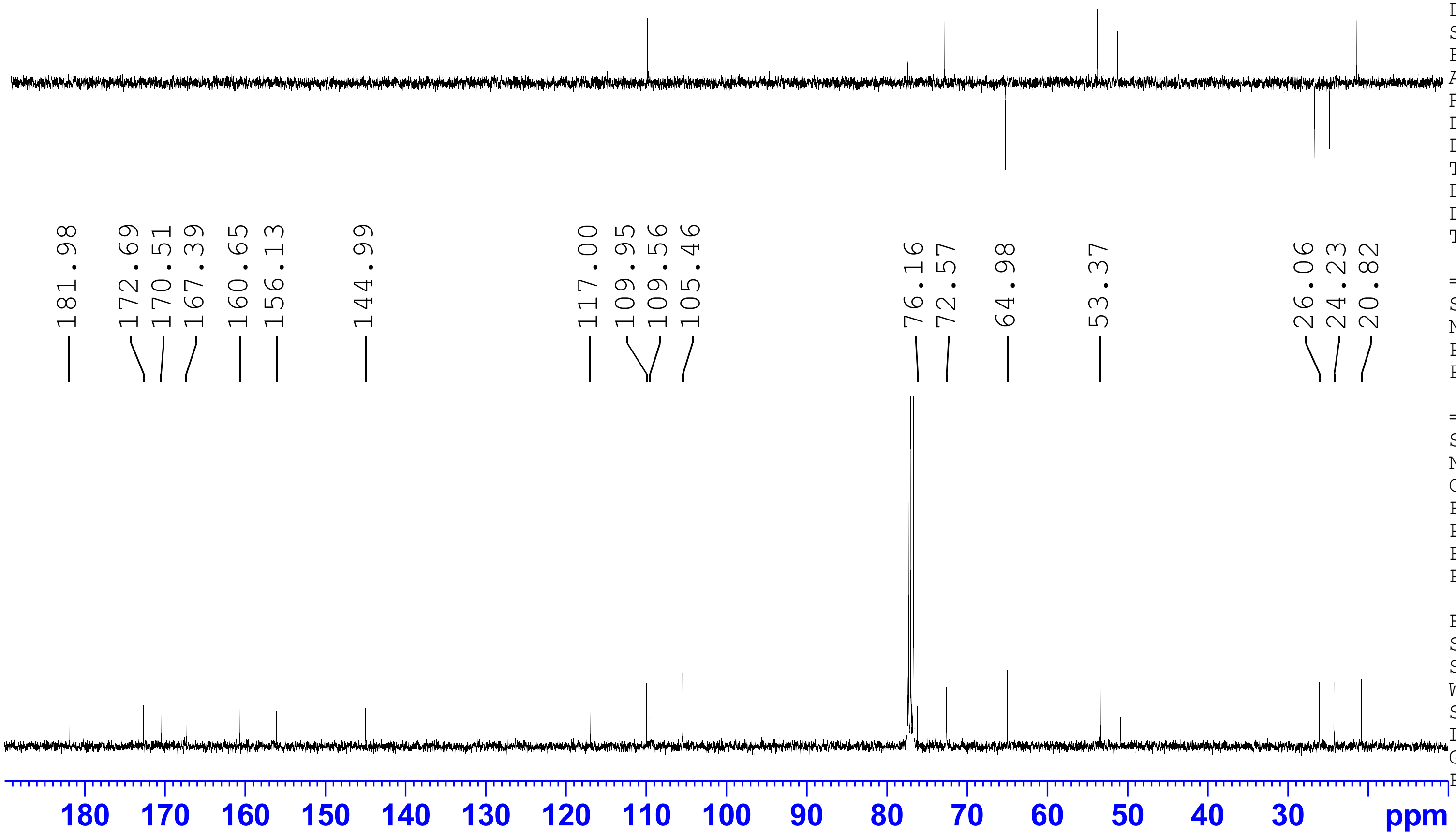

Current Data Parameters
NAME YJA-40 C 8mg
EXPNO 2
PROCNO 1

F2 - Acquisition Parameters
Date_ 20210912
Time 17.30
INSTRUM spect
PROBHD 5 mm PABBO BB-
PULPROG zgpg30
TD 65536
SOLVENT CDCl3
NS 484
DS 4
SWH 25252.525 Hz
FIDRES 0.385323 Hz
AQ 1.2976128 sec
RG 203
DW 19.800 usec
DE 6.50 usec
TE 296.5 K
D1 2.00000000 sec
D11 0.03000000 sec
TD0 1

======== CHANNEL f1 ========
SFO1 100.6228293 MHz
NUC1 13C
P1 12.37 usec
PLW1 28.13500023 W

======== CHANNEL f2 ========
SFO2 400.1316005 MHz
NUC2 1H
CPDPRG[2 waltz16
PCPD2 90.00 usec
PLW2 12.14299965 W
PLW12 0.28964999 W
PLW13 0.23461001 W

F2 - Processing parameters
SI 32768
SF 100.6127721 MHz
WDW EM
SSB 0
LB 1.00 Hz
GB 0
PC 1.40

Figure S2. ^{13}C NMR of compound **1**.

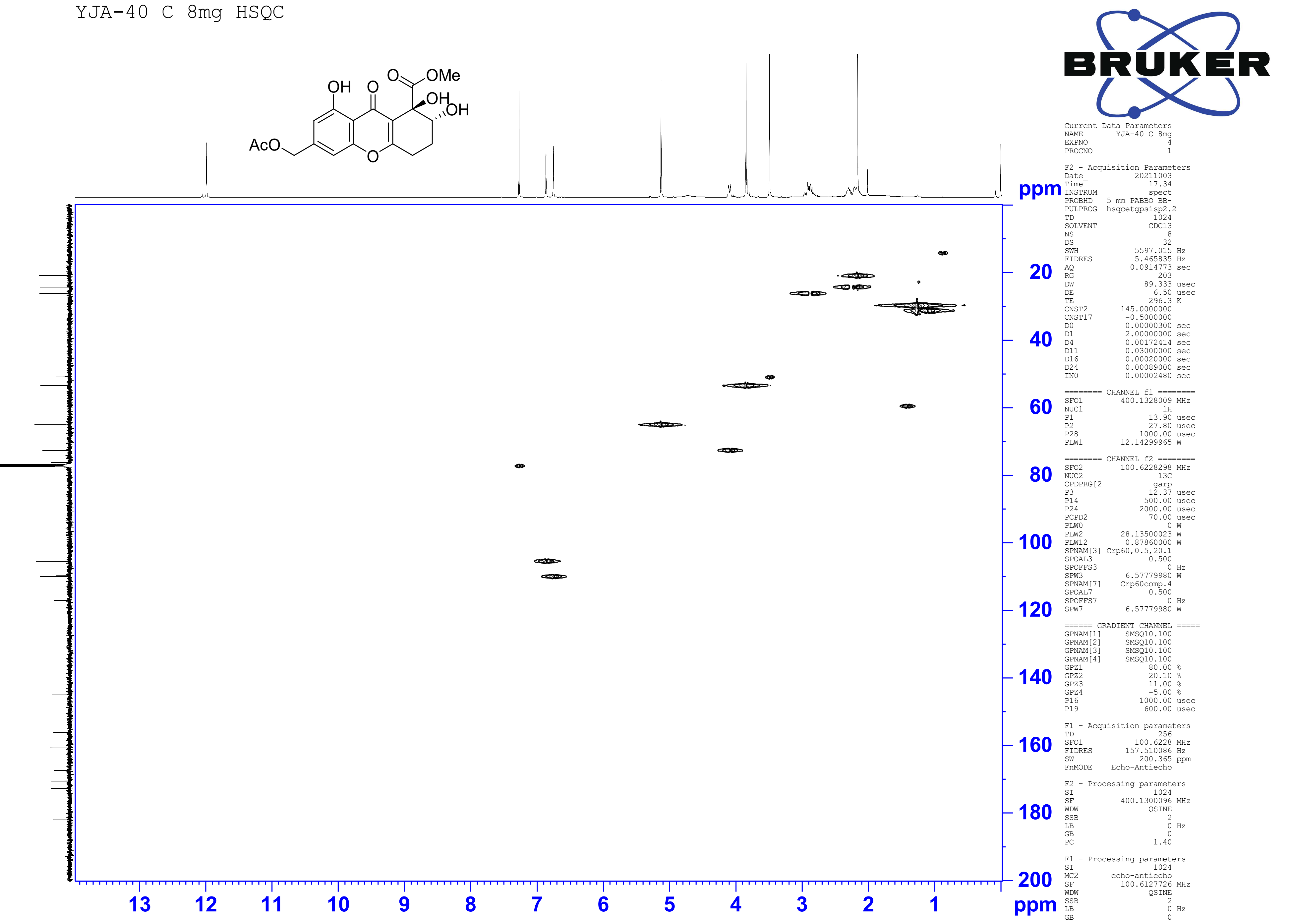

Figure S3. HSQC spectrum of compound **1**.

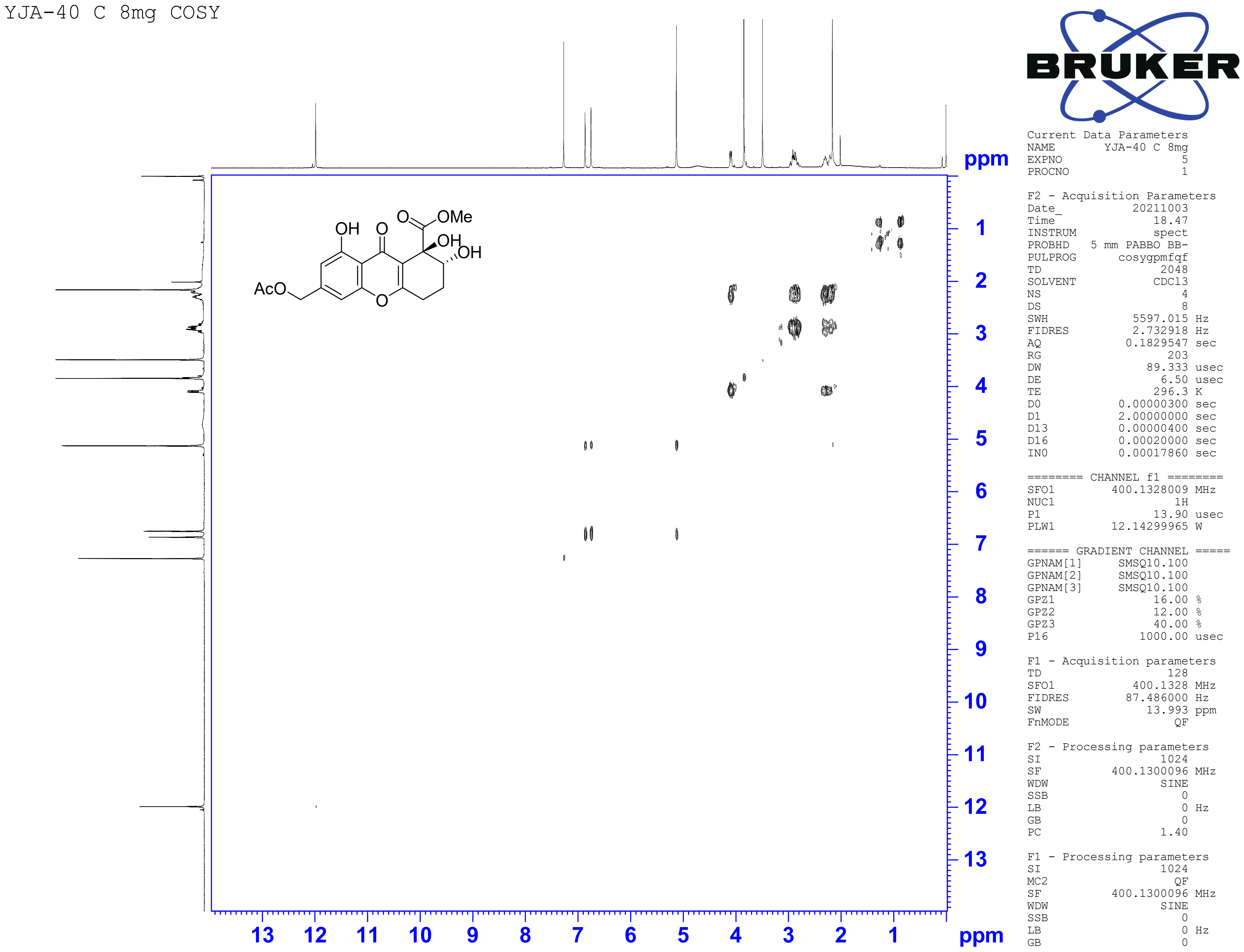

Figure S4. ^{1}H-^{1}H COSY spectrum of compound **1**.

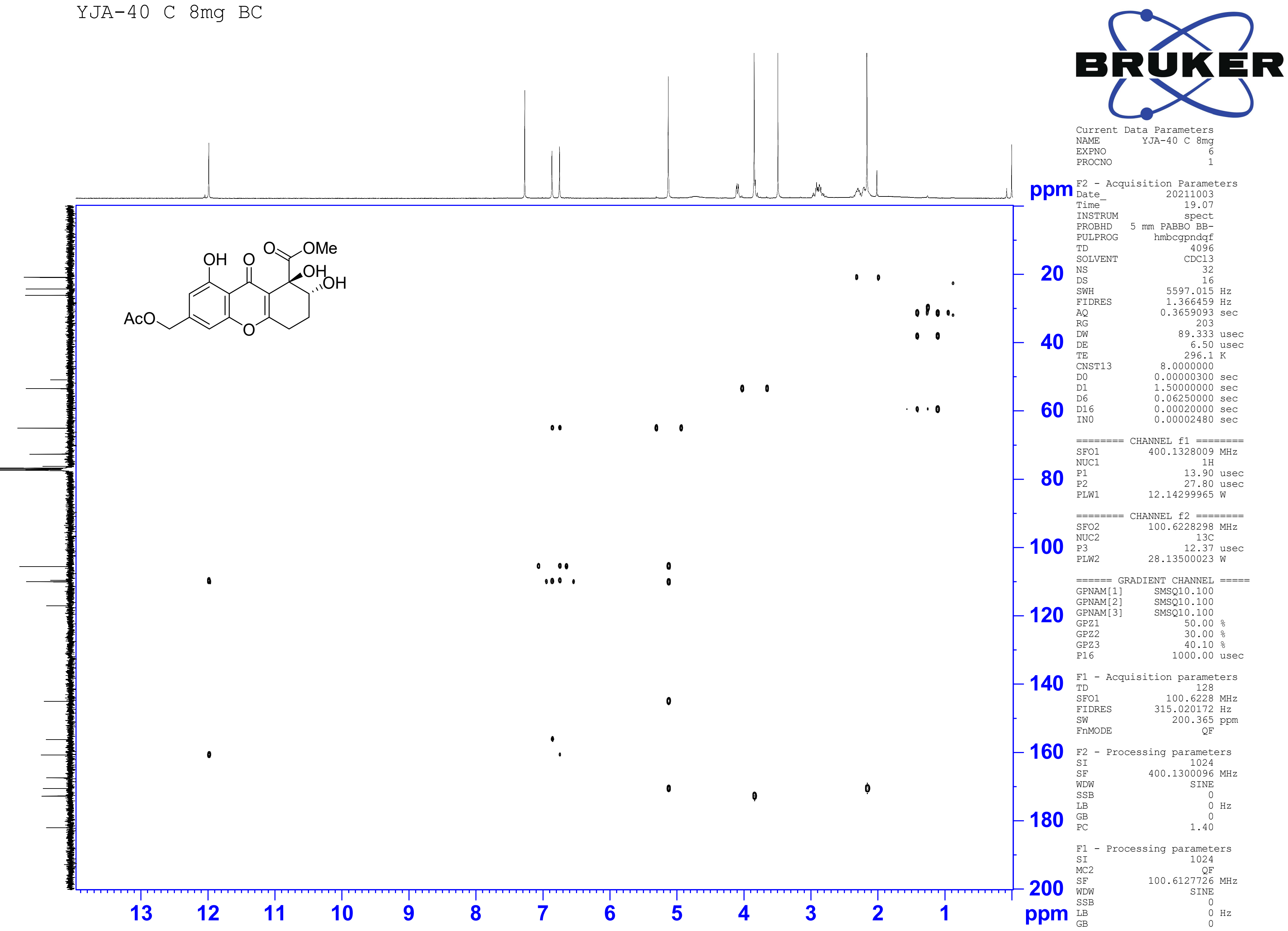

Figure S5. HMBC spectrum of compound **1**.

YJA-40 C 8mg NOESY

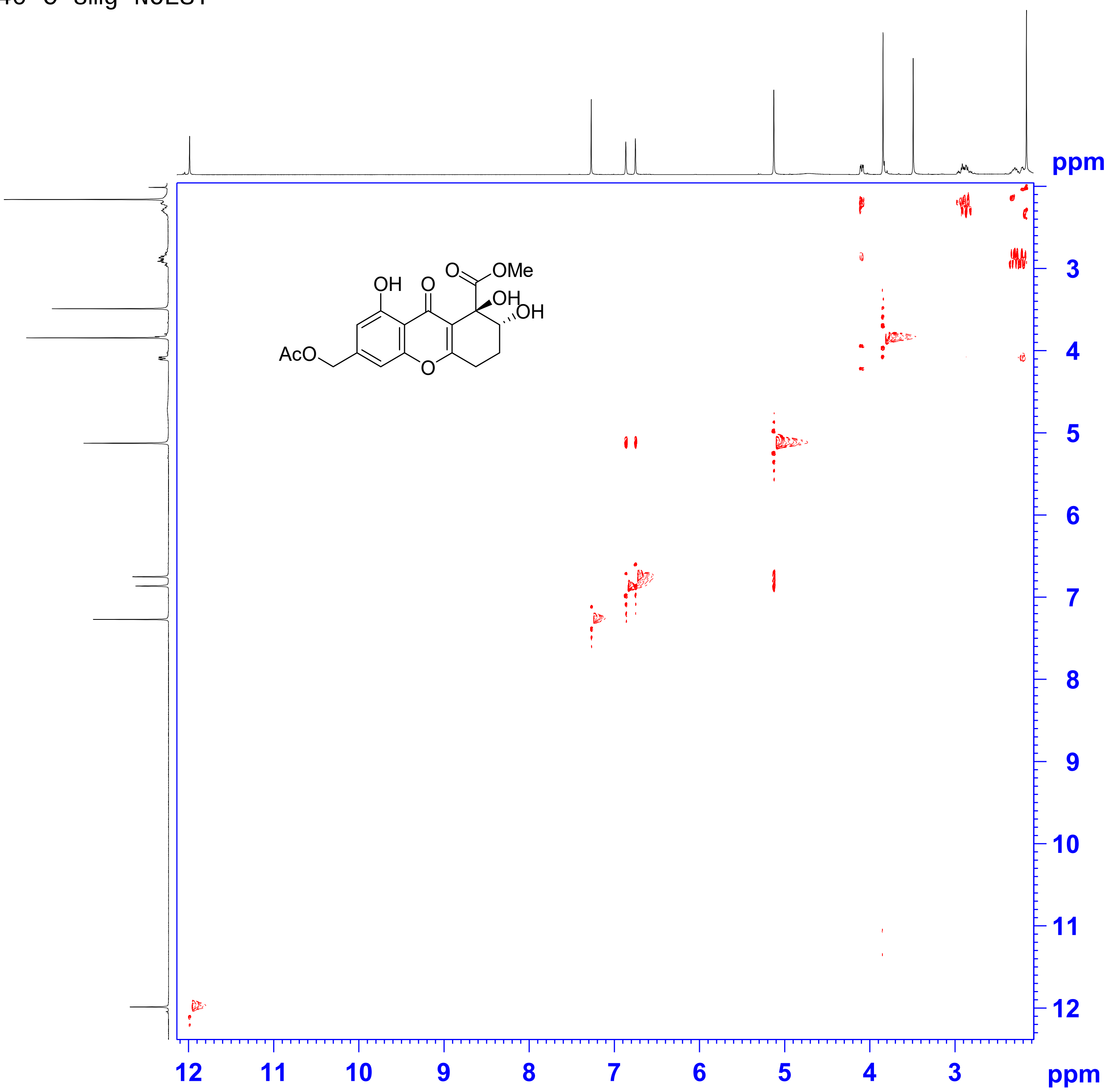

Current Data Parameters
NAME HYJ1-MD-1-YJA-40 C 8mg
EXPNO 7
PROCNO 1

F2 - Acquisition Parameters
Date_ 20220102
Time 18.46
INSTRUM spect
PROBHD 5 mm PABBO BB-
PULPROG noesygpphpp
TD 2048
SOLVENT CDCl3
NS 32
DS 32
SWH 5597.015 Hz
FIDRES 2.732918 Hz
AQ 0.1829547 sec
RG 203
DW 89.333 usec
DE 6.50 usec
TE 295.3 K
D0 0.00007160 sec
D1 2.00000000 sec
D8 0.30000001 sec
D11 0.03000000 sec
D12 0.00002000 sec
D16 0.00020000 sec
IN0 0.00017860 sec

======== CHANNEL f1 ========
SFO1 400.1328009 MHz
NUC1 1H
P1 13.90 usec
P2 27.80 usec
P17 2500.00 usec
PLW1 12.14299965 W
PLW10 3.47059989 W

====== GRADIENT CHANNEL =====
GPNAM[1] SMSQ10.100
GPZ1 40.00 %
P16 1000.00 usec

F1 - Acquisition parameters
TD 256
SFO1 400.1328 MHz
FIDRES 21.871500 Hz
SW 13.993 ppm
FnMODE States-TPPI

F2 - Processing parameters
SI 1024
SF 400.1300059 MHz
WDW QSINE
SSB 2
LB 0 Hz
GB 0
PC 1.00

F1 - Processing parameters
SI 1024
MC2 States-TPPI
SF 400.1300098 MHz
WDW QSINE
SSB 2
LB 0 Hz
GB 0

Figure S6. NOESY spectrum of compound 1.

Elemental Composition Report

Tolerance = 50.0 PPM / DBE: min = -1.5, max = 50.0
Element prediction: Off
Number of isotope peaks used for i-FIT = 3

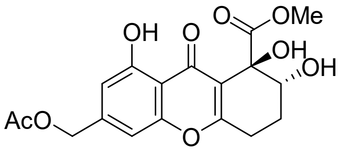

Monoisotopic Mass, Even Electron Ions
99 formula(e) evaluated with 9 results within limits (up to 50 best isotopic matches for each mass)
Elements Used:
C: 5-35 H: 0-60 O: 0-10 23Na: 0-1
YJA-40 71 (0.293) Cm (65:85)
1: TOF MS ES+

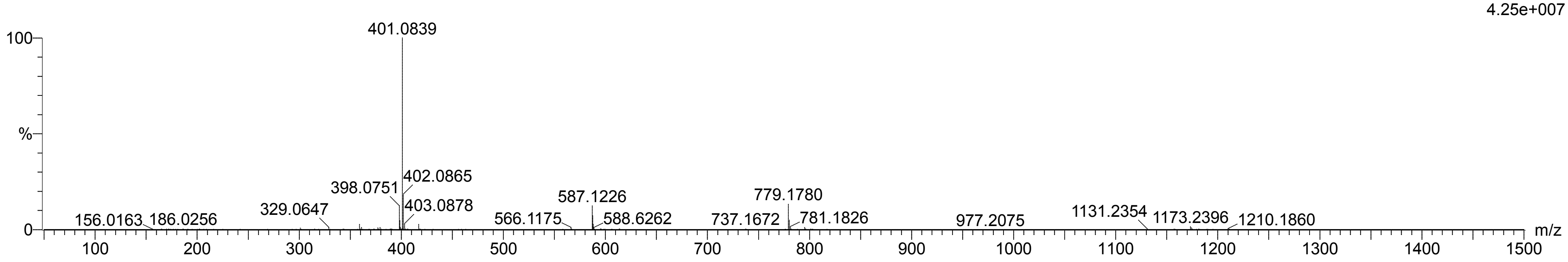

Minimum:	80.00				-1.5
Maximum:	100.00		50.0	50.0	50.0

Mass	RA	Calc. Mass	mDa	PPM	DBE	i-FIT	Norm	Conf(%)	Formula
401.0839	100.00	401.0849	-1.0	-2.5	9.5	1761.3	0.069	93.32	C18 H18 O9 23Na
		401.0873	-3.4	-8.5	12.5	1764.1	2.895	5.53	C20 H17 O9
		401.1001	-16.2	-40.4	13.5	1766.4	5.144	0.58	C22 H18 O6 23Na
		401.0661	17.8	44.4	17.5	1767.3	6.030	0.24	C23 H13 O7
		401.1025	-18.6	-46.4	16.5	1767.9	6.635	0.13	C24 H17 O6
		401.0790	4.9	12.2	18.5	1768.0	6.743	0.12	C25 H14 O4 23Na
		401.0814	2.5	6.2	21.5	1768.9	7.652	0.05	C27 H13 O4
		401.0942	-10.3	-25.7	22.5	1769.9	8.698	0.02	C29 H14 O 23Na
		401.0966	-12.7	-31.7	25.5	1770.6	9.377	0.01	C31 H13 O

Figure S7. HR-ESI-MS spectrum of compound **1**.

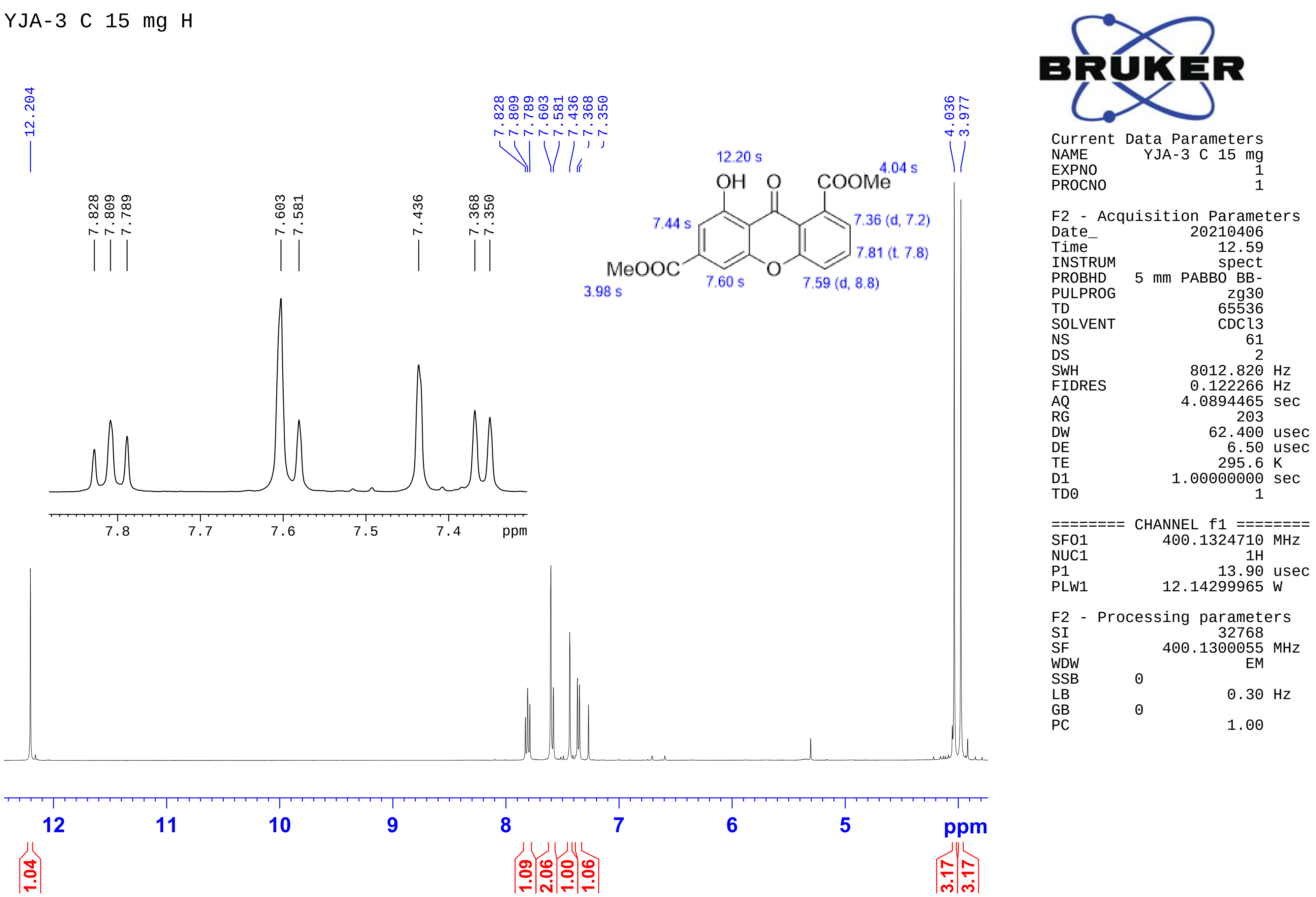

Figure S8. 1H NMR spectrum of compound **2**.

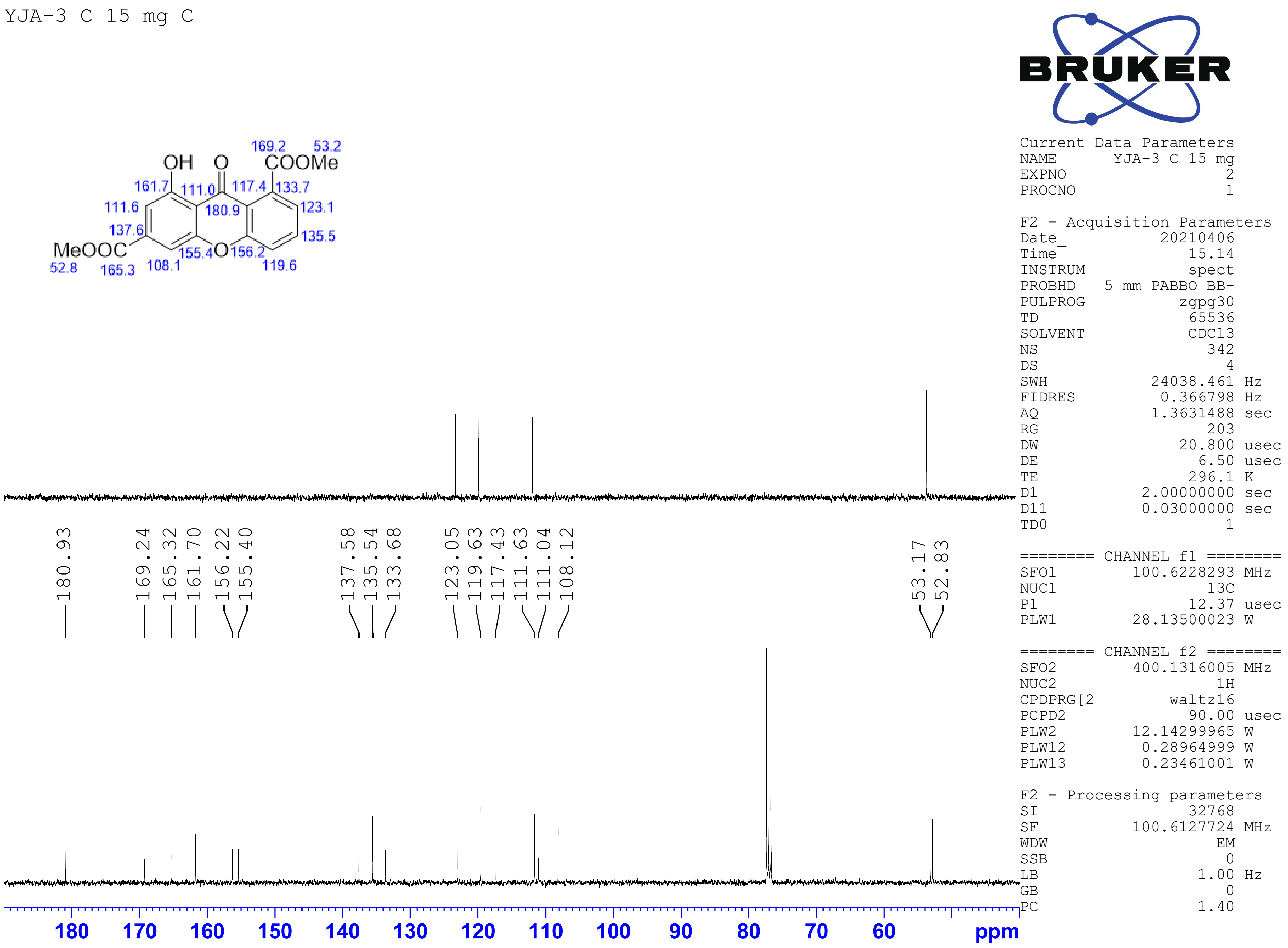

Figure S9. 13C NMR and DEPT of compound **2**.

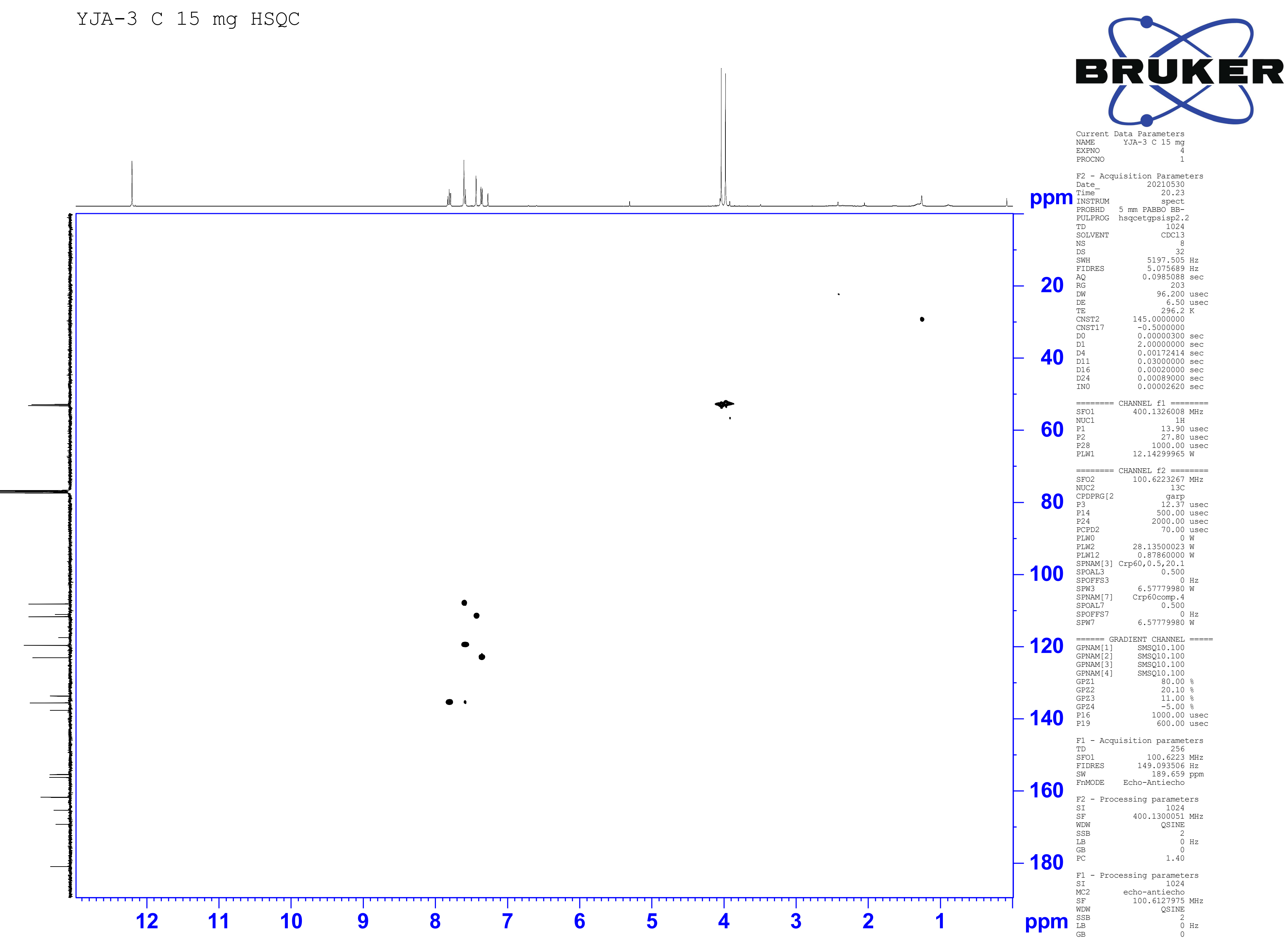

Figure S10. HMQC spectrum of compound **2**.

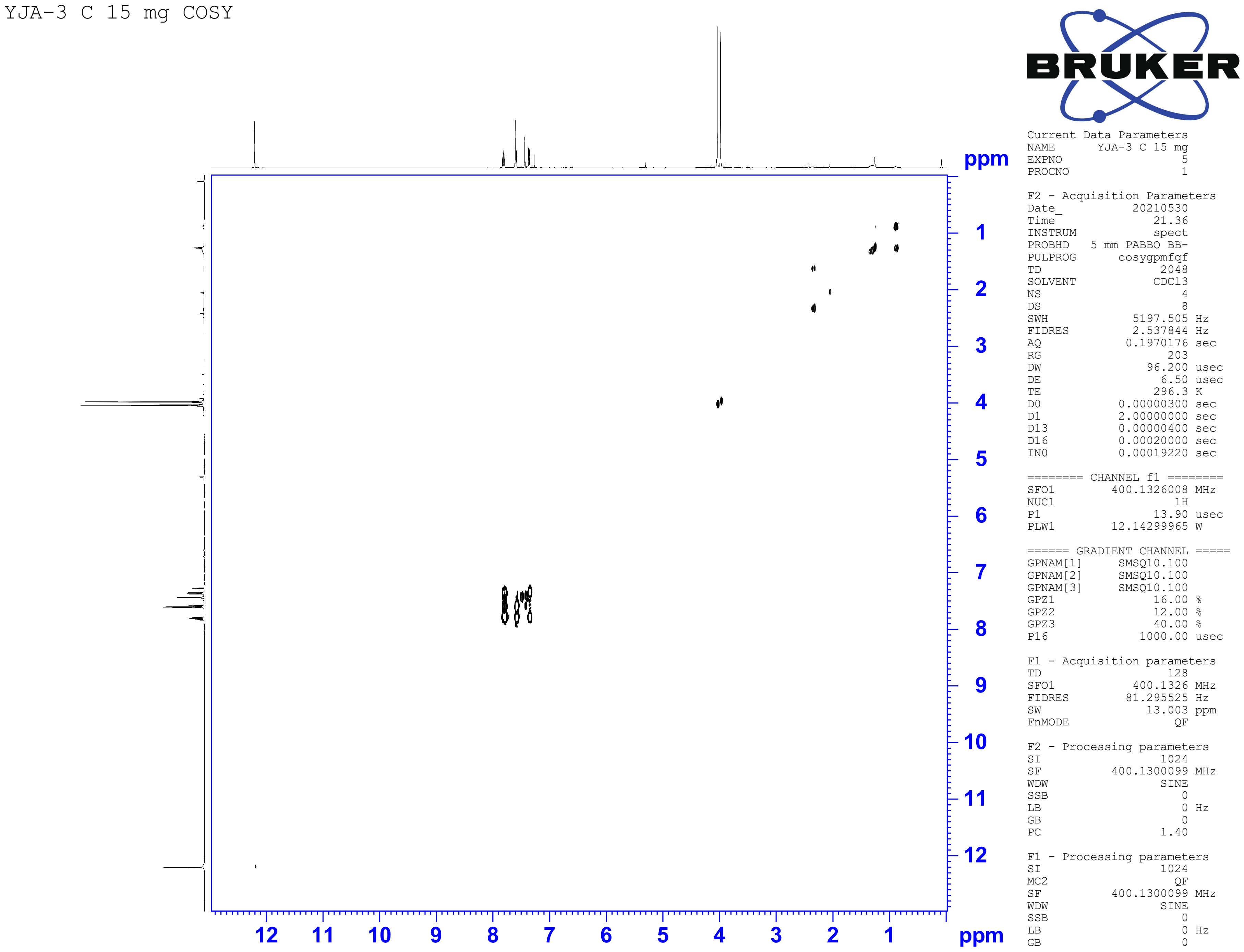

Figure S11. 1H-1H COSY spectrum of compound **2**.

YJA-3 C 15 mg HMBC

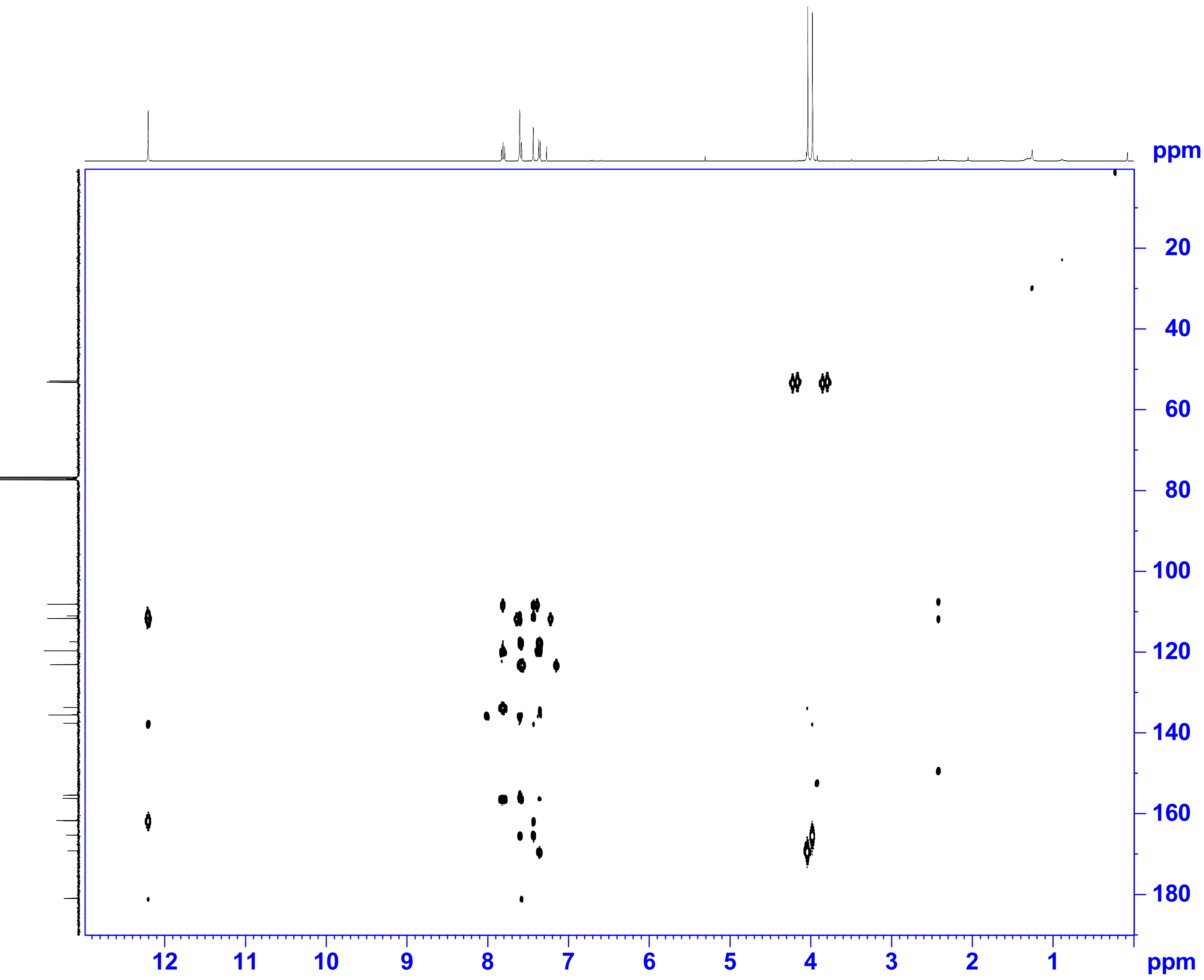

Current Data Parameters
NAME YJA-3 C 15 mg
EXPNO 6
PROCNO 1

F2 - Acquisition Parameters
Date_ 20210530
Time 21.57
INSTRUM spect
PROBHD 5 mm PABBO BB-
PULPROG hmbcgpndqf
TD 4096
SOLVENT CDCl3
NS 32
DS 16
SWH 5197.505 Hz
FIDRES 1.268922 Hz
AQ 0.3940352 sec
RG 203
DW 96.200 usec
DE 6.50 usec
TE 295.9 K
CNST13 8.0000000
D0 0.00000300 sec
D1 1.50000000 sec
D6 0.06250000 sec
D16 0.00020000 sec
IN0 0.00002620 sec

======== CHANNEL f1 ========
SFO1 400.1326008 MHz
NUC1 1H
P1 13.90 usec
P2 27.80 usec
PLW1 12.14299965 W

======== CHANNEL f2 ========
SFO2 100.6223267 MHz
NUC2 13C
P3 12.37 usec
PLW2 28.13500023 W

====== GRADIENT CHANNEL =====
GPNAM[1] SMSQ10.100
GPNAM[2] SMSQ10.100
GPNAM[3] SMSQ10.100
GPZ1 50.00 %
GPZ2 30.00 %
GPZ3 40.10 %
P16 1000.00 usec

F1 - Acquisition parameters
TD 128
SFO1 100.6223 MHz
FIDRES 298.187012 Hz
SW 189.659 ppm
FnMODE QF

F2 - Processing parameters
SI 1024
SF 400.1300033 MHz
WDW SINE
SSB 0
LB 0 Hz
GB 0
PC 1.40

F1 - Processing parameters
SI 1024
MC2 QF
SF 100.6127437 MHz
WDW SINE
SSB 0
LB 0 Hz
GB 0

Figure S12. HMBC spectrum of compound **2**.

Elemental Composition Report

Tolerance = 50.0 PPM / DBE: min = -1.5, max = 50.0
Element prediction: Off
Number of isotope peaks used for i-FIT = 3

Monoisotopic Mass, Even Electron Ions
91 formula(e) evaluated with 8 results within limits (up to 50 best isotopic matches for each mass)
Elements Used:
C: 5-35 H: 0-60 O: 0-10 23Na: 0-1
YJA-3 95 (0.379) Cm (79:114)
1: TOF MS ES+

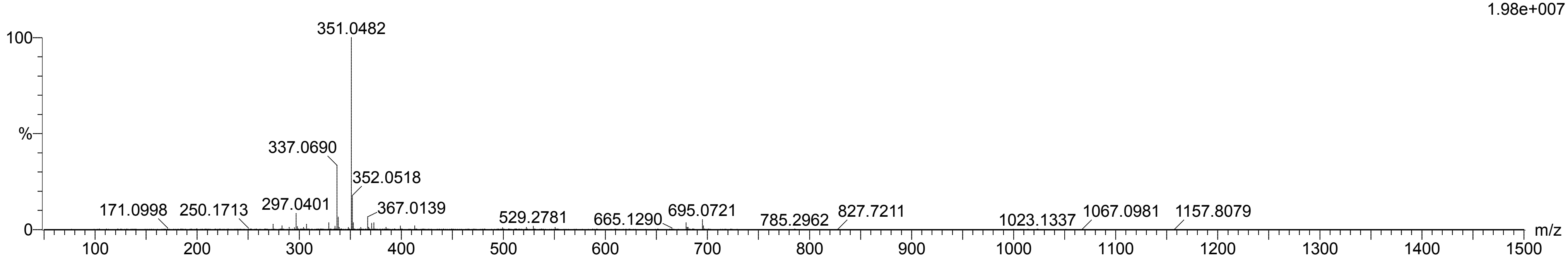

Minimum:	80.00				-1.5				
Maximum:	100.00		50.0	50.0	50.0				

Mass	RA	Calc. Mass	mDa	PPM	DBE	i-FIT	Norm	Conf(%)	Formula
351.0482	100.00	351.0352	13.0	37.0	10.5	2070.7	0.155	85.64	C15 H11 O10
		351.0481	0.1	0.3	11.5	2072.8	2.247	10.57	C17 H12 O7 23Na
		351.0328	15.4	43.9	7.5	2074.3	3.814	2.20	C13 H12 O10 23Na
		351.0505	-2.3	-6.6	14.5	2074.8	4.271	1.40	C19 H11 O7
		351.0633	-15.1	-43.0	15.5	2077.3	6.727	0.12	C21 H12 O4 23Na
		351.0657	-17.5	-49.9	18.5	2078.5	7.969	0.03	C23 H11 O4
		351.0422	6.0	17.1	20.5	2079.1	8.587	0.02	C24 H8 O2 23Na
		351.0446	3.6	10.3	23.5	2079.9	9.370	0.01	C26 H7 O2

Figure S13. HR-ESI-MS spectrum of compound **2**.
